# Supplementary material for: Short-Chained Alcohols Make Membrane Surfaces Conducive for Melittin Action: Implication for the Physiological Role of Alcohols in Cells
Source: Cells. 2022 Jun 15;11(12):1928. doi: 10.3390/cells11121928 (PMC9221640; doi:10.3390/cells11121928)
Supplement: Supplementary file 1 [file cells-11-01928-s001.zip › cells-1731763-supplementary.pdf]

Appendix

Article

Short-Chained Alcohols Make Membrane Surface Conducive for Melittin Action: Implication for Physiological Role of Alcohols in Cells

Haoyu Wang, Hao Qin, Győző Garab and Edward S. Gasanoff

Table S1. Effects of alcohols (1.65×10^{-3} M) and melittin (1.65×10^{-4} M) on the heart rate of *Daphnia pulex*.

Number of carbons in alcohol	**0** no alcohol	**1**	**2**	**3**	**4**	**5**	**6**	**7**	**8**	**9**	**10**	Anova test *p*-values
**Heart beats per minute** (bpm) alcohol only	269<br>263<br>267	260<br>262<br>257	202<br>201<br>197	177<br>181<br>182	170<br>169<br>171	158<br>154<br>162	146<br>143<br>149	140<br>136<br>143	128<br>130<br>132	198<br>193<br>191	225<br>221<br>220	0.00000
Means	266	260	200	180	170	158	146	140	130	194	222	–
SD	± 3.1	± 2.5	± 2.6	± 2.6	± 1.0	± 4.0	± 3.0	± 3.5	± 2.0	± 3.6	± 2.6	–
**Heart beats per minute** (bpm) alcohol+melittin	285<br>288<br>284	285<br>289<br>281	324<br>324<br>327	342<br>337<br>346	349<br>349<br>352	356<br>356<br>353	360<br>362<br>358	367<br>363<br>362	370<br>369<br>365	311<br>306<br>315	296<br>300<br>291	0.00000
Means	286	285	325	341	350	355	360	364	368	311	296	–
SD	± 2.1	± 4.0	± 1.7	± 4.5	± 1.7	± 1.7	± 2.0	± 2.6	± 2.6	± 4.5	± 4.5	–
p-values*	0.00145	0.00163	0.00000	0.00001	0.00000	0.00001	0.00000	0.00000	0.00000	0.00001	0.00009	–

**p*-values for statistical comparison between series of experiments with "alcohols only" against "alcohols+melittin" are calculated to 5 decimal places. Anova test *p*-values are also calculated to 5 decimal places.

Table S2. Effects of alcohols (1.65 × 10^{-3} M) and melittin (1.65 × 10^{-4} M) on the *Daphnia pulex* mitochondria ATP level. The phospholipid concentration in mitochondrial samples assessed by normalizing the integrated area of the ^{31}P NMR signals to that of large multilamellar liposomes was approximately 1.65 × 10^{-2} M.

Number of carbons in alcohol	**0** no alcohol	**1**	**2**	**3**	**4**	**5**	**6**	**7**	**8**	**9**	**10**	Anova test *p*-values
**µmol ATP per mg of protein** alcohol only	1.73<br>1.69<br>1.76	1.72<br>1.68<br>1.64	1.37<br>1.42<br>1.41	1.35<br>1.29<br>1.35	1.24<br>1.29<br>1.25	1.19<br>1.17<br>1.21	1.11<br>1.11<br>1.14	1.07<br>1.04<br>1.04	0.97<br>0.97<br>1.00	1.45<br>1.49<br>1.42	1.57<br>1.54<br>1.52	0.00000
Means	1.73	1.68	1.40	1.33	1.26	1.19	1.12	1.05	0.98	1.45	1.54	–
SD	± 0.035	± 0.040	± 0.026	± 0.035	± 0.026	± 0.020	± 0.017	± 0.017	± 0.017	± 0.035	± 0.025	–
**µmol ATP per mg of protein** alcohol+melittin	1.86<br>1.82<br>1.81	1.87<br>1.91<br>1.86	2.89<br>2.88<br>2.84	3.27<br>3.29<br>3.34	3.57<br>3.61<br>3.62	3.85<br>3.93<br>3.86	4.03<br>4.04<br>4.08	4.15<br>4.14<br>4.07	4.25<br>4.12<br>4.20	2.70<br>2.69<br>2.80	2.42<br>2.33<br>2.45	0.00000
Means	1.83	1.88	2.87	3.30	3.60	3.88	4.05	4.12	4.19	2.73	2.40	–
SD	± 0.026	± 0.026	± 0.026	± 0.036	± 0.026	± 0.044	± 0.026	± 0.044	± 0.066	± 0.061	± 0.062	–
p-values*	0.01763	0.00331	0.00000	0.00000	0.00000	0.00001	0.00000	0.00001	0.00005	0.00004	0.00046	–

**p*-values for statistical comparison between series of experiments with "alcohols only" against "alcohols+melittin" were calculated to 5 decimal places. Anova test *p*-values are also calculated to 5 decimal places.

Table S3. Effects of alcohols (5.5×10^{-4} M) and melittin (5.5×10^{-5} M) on the B/C ratio of the EPR spectra of 5-DSA in oriented lipid films. The concentration of phospholipids and 5-DSA in lipid films was 5.5×10^{-3} M and 5.5×10^{-4} M respectively.

Number of carbons in alcohol	**0** no alcohol	**1**	**2**	**3**	**4**	**5**	**6**	**7**	**8**	**9**	**10**	Anova test *p*-values
**B/C ratio** alcohol only raw data	0.64<br>0.67<br>0.64	0.65<br>0.62<br>0.65	0.57<br>0.56<br>0.55	0.56<br>0.53<br>0.54	0.52<br>0.53<br>0.51	0.49<br>0.50<br>0.51	0.47<br>0.49<br>0.48	0.46<br>0.45<br>0.47	0.44<br>0.46<br>0.45	0.59<br>0.58<br>0.57	0.61<br>0.62<br>0.60	0.00000
Means	0.65	0.64	0.56	0.54	0.52	0.50	0.48	0.46	0.45	0.58	0.61	–
SD	± 0.017	± 0.017	± 0.010	± 0.015	± 0.010	± 0.010	± 0.010	± 0.010	± 0.010	± 0.010	± 0.010	–
**B/C ratio** alcohol+melittin raw data	0.4589<br>0.4701<br>0.4810	0.4600<br>0.4510<br>0.4690	0.3697<br>0.3610<br>0.3793	0.3585<br>0.3499<br>0.3416	0.3299<br>0.3376<br>0.3225	0.3099<br>0.3026<br>0.3175	0.2901<br>0.2965<br>0.2834	0.2765<br>0.2700<br>0.2635	0.2663<br>0.2601<br>0.2536	0.4208<br>0.4301<br>0.4391	0.4409<br>0.4499<br>0.4592	0.00000
Means	0.4700	0.4600	0.3700	0.3500	0.3300	0.3100	0.2900	0.2700	0.2600	0.4300	0.4500	–
SD	± 0.0111	± 0.0090	± 0.0092	± 0.0085	± 0.0076	± 0.0075	± 0.0066	± 0.0065	± 0.0064	± 0.0092	± 0.0092	–
p-values*	0.00031	0.00053	0.00002	0.00024	0.00002	0.00002	0.00004	0.00004	0.00004	0.00005	0.00004	–

**p*-values for statistical comparison between series of experiments with "alcohols only" against "alcohols+melittin" are calculated to 5 decimal places. Anova test *p*-values are also calculated to 5 decimal places.

Table S4. Effects of alcohols (5.5×10^{-4} M) and melittin (6.11×10^{-5} M) on the parameter *S* of the EPR spectra of 5-DSA in oriented lipid films. The concentration of phospholipids and 5-DSA in lipid films was 5.5×10^{-3} M and 5.5×10^{-4} M respectively.

Number of carbons in alcohol	**0** no alcohol	**1**	**2**	**3**	**4**	**5**	**6**	**7**	**8**	**9**	**10**	Anova test *p*-values
**Parameter *S*** alcohol only raw data	0.68<br>0.71<br>0.74	0.70<br>0.73<br>0.67	0.53<br>0.58<br>0.55	0.55<br>0.53<br>0.51	0.53<br>0.51<br>0.49	0.49<br>0.51<br>0.47	0.49<br>0.47<br>0.45	0.46<br>0.43<br>0.49	0.42<br>0.45<br>0.48	0.62<br>0.59<br>0.65	0.67<br>0.63<br>0.65	0.00000
Means	0.71	0.70	0.55	0.53	0.51	0.49	0.47	0.46	0.45	0.62	0.65	–
SD	0.03	0.03	0.03	0.02	0.02	0.02	0.02	0.02	0.02	0.03	0.02	–
**Parameter *S*** alcohol + melittin raw data	0.81<br>0.84<br>0.78	0.85<br>0.79<br>0.82	0.91<br>0.89<br>0.93	0.91<br>0.95<br>0.93	0.93<br>0.95<br>0.97	0.99<br>0.95<br>0.97	0.99<br>1.01<br>0.97	1.01<br>0.99<br>1.03	1.05<br>1.01<br>1.03	0.86<br>0.88<br>0.84	0.86<br>0.84<br>0.82	0.00000
Means	0.81	0.82	0.91	0.93	0.95	0.97	0.99	1.01	1.03	0.86	0.84	–
SD	0.03	0.03	0.02	0.02	0.02	0.02	0.02	0.02	0.02	0.02	0.02	–
p-values*	0.01507	0.00805	0.00006	0.00002	0.00001	0.00001	0.00001	0.00004	0.00003	0.00067	0.00031	–

**p*-values for statistical comparison between series of experiments with "alcohols only" against "alcohols+melittin" are calculated to 5 decimal places. Anova test *p*-values are also calculated to 5 decimal places.

Table S5. Increase in membrane permeability to $Fe(CN)_6^{3-}$ ions in the membranes of sonicated unilamellar liposomes treated with melittin and alcohols. The permeability is assessed by calculating the I_i/I_o ratio, where I_i and I_o are, respectively, the inner and the outer leaflet ^{1}H-NMR PC signal areas. The total phospholipid concentration was 1.2 ×10^{-2} M. In all experiments the alcohol concentration was 1.2 ×10^{-3} M, while melittin concentration was 1.2 ×10^{-4} M or 1.2 ×10^{-3} M in different series of experiments. Each data point is the mean of three independent experiments. Standard deviations for all data points were within 4.5% of the means. Abbreviations: **M – melittin, met** – methanol, **eth** – ethanol, **pro** – propanol, **but** – butanol, **pent** – pentanol, **hex** – hexanol, **hep** – heptanol, **oct** – octanol, **non** – nonanol, **dec** – decanol.

**1.2×10^{-4} M**	**Control**	**M**	**M+met**	**M+eth**	**M+pro**	**M+but**	**M+pent**	**M+hex**	**M+hep**	**M+oct**	**M+non**	**M+dec**	Anova<br>p-value
**I_i/I_o**<br>raw data	0.529<br>0.510<br>0.533	0.586<br>0.572<br>0.573	0.579<br>0.569<br>0.580	0.565<br>0.579<br>0.590	0.584<br>0.567<br>0.574	0.568<br>0.581<br>0.585	0.580<br>0.575<br>0.573	0.569<br>0.574<br>0.582	0.570<br>0.573<br>0.579	0.575<br>0.568<br>0.564	0.581<br>0.559<br>0.576	0.567<br>0.581<br>0.565	0.000004
**means**	**0.524**	**0.577**	**0.576**	**0.578**	**0.575**	**0.578**	**0.576**	**0.575**	**0.574**	**0.569**	**0.572**	**0.571**	—
SD	± 0.012	± 0.008	± 0.006	± 0.013	± 0.009	± 0.009	± 0.004	± 0.007	± 0.005	± 0.006	± 0.012	± 0.009	—
**1.2×10^{-3} M**	**Control**	**M**	**M+met**	**M+eth**	**M+pro**	**M+but**	**M+pent**	**M+hex**	**M+hep**	**M+oct**	**M+non**	**M+dec**	Anova<br>p-value
**I_i/I_o**<br>raw data	0.529<br>0.510<br>0.533	0.180<br>0.185<br>0.181	0.182<br>0.179<br>0.185	0.185<br>0.179<br>0.179	0.180<br>0.178<br>0.185	0.183<br>0.177<br>0.183	0.178<br>0.182<br>0.183	0.184<br>0.180<br>0.179	0.178<br>0.181<br>0.184	0.182<br>0.181<br>0.180	0.178<br>0.184<br>0.184	0.183<br>0.183<br>0.180	0.000000
**means**	**0.524**	**0.182**	**0.182**	**0.181**	**0.181**	**0.181**	**0.181**	**0.181**	**0.181**	**0.181**	**0.182**	**0.182**	—
SD	± 0.012	± 0.003	± 0.003	± 0.003	± 0.004	± 0.003	± 0.003	± 0.003	± 0.003	± 0.001	± 0.003	± 0.002	—
p-values*	1.00000	0.000027	0.000003	0.000132	0.000016	0.000024	0.000000	0.000009	0.000000	0.000042	0.000097	0.000098	—

*p-values for statistical comparison between series of experiments with melittin concentration 1.2 ×10^{-4} M against melittin concentration 1.2 ×10^{-3} M are calculated to 6 decimal places. Anova test p-values are also calculated to 6 decimal places.

Table S6. Formation of non-bilayer organized PC in the membranes of sonicated unilamellar liposomes treated with melittin and alcohols. The molar percentage of non-bilayer organized PC molecules was assessed by calculating the percentage of computer-extrapolated area under the ^{1}H-NMR non-bilayer signal from the overall area of the ^{1}H-NMR spectrum including signals from I_o, I_i and non-bilayer organized PC molecules using Gaussian fitting as previously described [22,29,36,41]. The total phospholipid concentration was 1.2 $\times 10^{-2}$ M. In all experiments the alcohol concentration was 1.2 $\times 10^{-3}$ M, while melittin concentration was 1.2 $\times 10^{-4}$ M or 1.2 $\times 10^{-3}$ M in different series of experiments. Each data point is the mean of three independent experiments. Standard deviations for all data points were within 4.7% of the means. Abbreviations: **M – melittin, met** – methanol, **eth** – ethanol, **pro** – propanol, **but** – butanol, **pent** – pentanol, **hex** – hexanol, **hep** – heptanol, **oct** – octanol, **non** – nonanol, **dec** – decanol, **NB (%)** – molar percentage of non-bilayer organized PC.

**1.2 × 10^{-4} M**	**Control**	**M**	**M+met**	**M+eth**	**M+pro**	**M+but**	**M+pent**	**M+hex**	**M+hep**	**M+oct**	**M+non**	**M+dec**	Anova *p*-value
**NB %** raw data	—	—	—	13.9<br>14.5<br>14.5	15.7<br>16.4<br>16.2	18.5<br>17.7<br>18.1	20.3<br>19.8<br>19.6	21.5<br>21.8<br>21.5	23.8<br>22.9<br>23.5	24.9<br>25.4<br>25.0	—	—	0.000000
**means**	**—**	**—**	**—**	**14.3**	**16.1**	**18.1**	**19.9**	**21.6**	**23.4**	**25.1**	**—**	**—**	**—**
SD	—	—	—	± 0.35	± 0.36	± 0.40	± 0.36	± 0.17	± 0.46	± 0.26	—	—	—
**1.2 × 10^{-3} M**	**Control**	**M**	**M+met**	**M+eth**	**M+pro**	**M+but**	**M+pent**	**M+hex**	**M+hep**	**M+oct**	**M+non**	**M+dec**	Anova *p*-value
**NB %** raw data	—	36.0<br>37.7<br>37.0	37.6<br>36.2<br>36.9	36.1<br>37.8<br>37.1	37.7<br>36.1<br>37.2	37.2<br>36.2<br>37.6	36.4<br>37.8<br>36.8	37.5<br>37.1<br>36.4	36.0<br>37.5<br>37.5	37.2<br>37.5<br>36.3	36.3<br>37.8<br>36.6	37.6<br>37.0<br>36.1	0.999999
**means**	**—**	**36.9**	**36.9**	**37.0**	**37.0**	**37.0**	**37.0**	**37.0**	**37.0**	**37.0**	**36.9**	**36.9**	**—**
SD	—	± 0.85	± 0.70	± 0.85	± 0.82	± 0.72	± 0.72	± 0.56	± 0.87	± 0.62	± 0.79	± 0.75	—
p-values*	—	—	—	0.000080	0.000068	0.000025	0.000052	0.000145	0.000144	0.000170	—	—	—

**p*-values for statistical comparison between series of experiments with melittin concentration 1.2 $\times 10^{-4}$ M against melittin concentration 1.2 $\times 10^{-3}$ M are calculated to 6 decimal places. Anova test *p*-values are also calculated to 6 decimal places.
